# Artifact reduction with a wideband late gadolinium enhancement (LGE) MRI technique for patients with implanted cardiac devices: a two-center study

**DOI:** 10.1186/1532-429X-16-S1-O29

**Published:** 2014-01-16

**Authors:** Shams Rashid, Adam Plotnik, Harold Litt, Yuchi Han, Stanislas Rapacchi, Roderick H Tung, Kalyanam Shivkumar, J Paul Finn, Peng Hu

**Affiliations:** 1 Department of Radiological Sciences, University of California, Los Angeles, Los Angeles, California, USA; 2 Department of Radiology, University of Pennsylvania Perelman School of Medicine, Philadelphia, Pennsylvania, USA; 3 Department of Medicine, University of Pennsylvania Perelman School of Medicine, Philadelphia, Pennsylvania, USA; 4 Penn Cardiovascular Institute, University of Pennsylvania Perelman School of Medicine, Philadelphia, Pennsylvania, USA; 5 UCLA Cardiac Arrhythmia Center, UCLA Health System, Los Angeles, California, USA; 6 Biomedical Physics Inter-Departmental Graduate Program, University of California, Los Angeles, Los Angeles, California, USA

## Background

**Figure 1:**
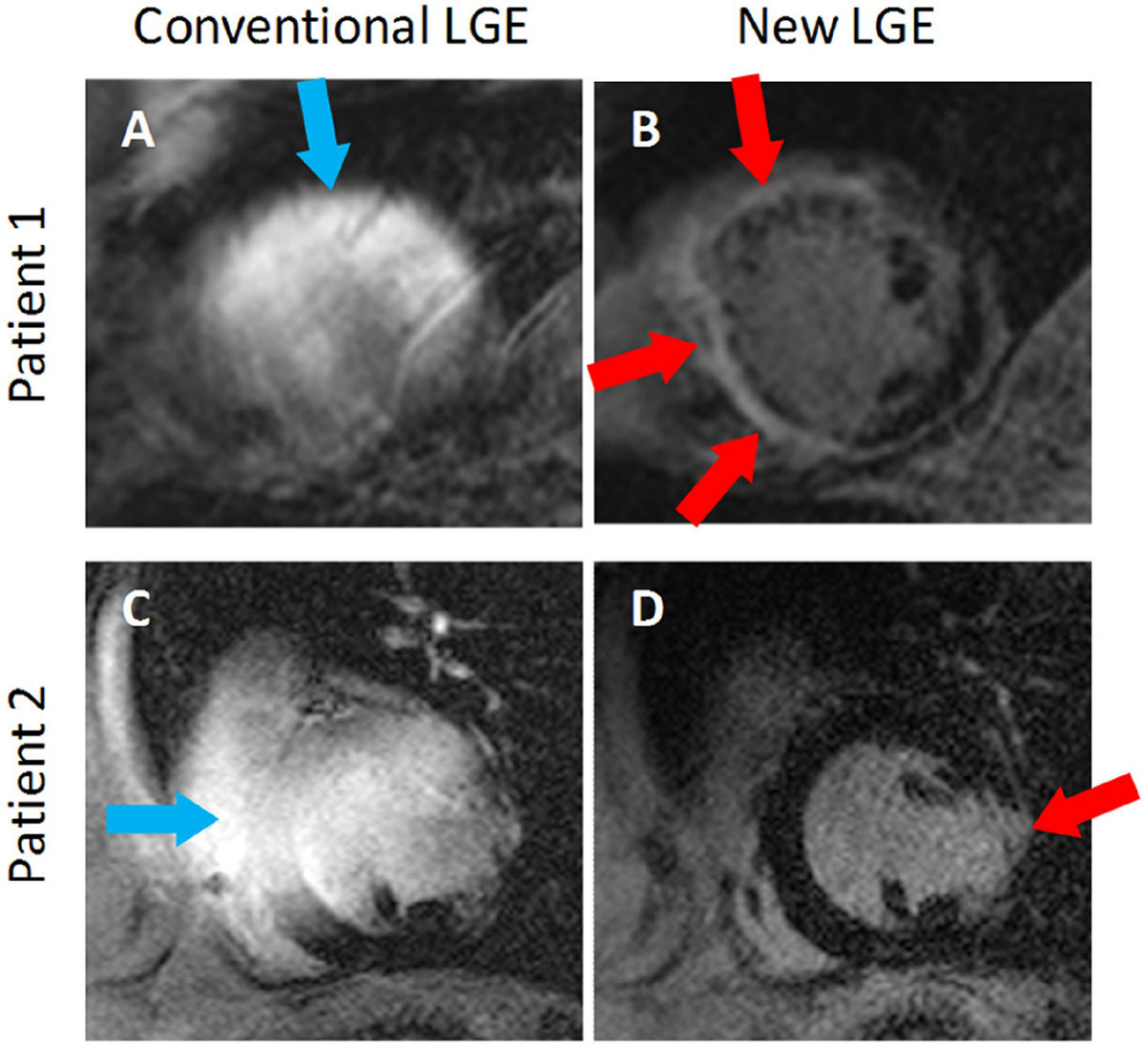
**Examples of conventional LGE (A & C) and wideband LGE (B & D) images from two patients**. In both patients, severe hyper-intensity artifacts (blue arrows) are produced in the conventional sequence, which prevents assessment of scar. The artifacts are completely eliminated by the wideband LGE technique. Scar tissue (red arrows) was identified in the anterior and septal walls of the left ventricle in Patient 1, and in the lateral wall of the left ventricle in Patient 2.

Late gadolinium enhancement (LGE) cardiac MRI is the clinical gold standard for non-invasive characterization of myocardial scar [1]. However, up to 75% of patients who may benefit from LGE MRI have preexisting implanted cardiac devices such as implantable cardioverter defibrillators (ICD) and pacemakers (PM) [2]. The presence of an ICD produces hyper-intensity (HI) image artifacts in LGE (Figure 1) and can prevent assessment of myocardial scar. We recently proposed a wideband LGE MRI technique that removes these artifacts in ICD patients [3,4]. In this abstract, we present our two-center experience of using this wideband LGE sequence on a cohort of patients with ICDs who were referred to cardiac MRI.

## Methods

The HI artifacts in LGE images of ICD patients are caused by severe off-resonance produced by the ICD. Spins in the affected myocardium are not inverted by the IR pulse and give rise to the HI artifacts. In the new sequence, a wideband (3.8 kHz) IR pulse was implemented to replace the standard pulse with 1.1 kHz spectral bandwidth, thereby eliminating the HI artifacts [3,4]. The wideband LGE was implemented at the medical centers of the University of California, Los Angeles (UCLA), and the University of Pennsylvania (Penn). A total of 25 patients with ICDs and PMs (UCLA: 19, Penn: 6), were imaged using the conventional and the wideband LGE technique. In each image set, the left ventricle was divided into 13 segments (basal, mid-ventricular, and apical, each having posterior, lateral, anterior and septal segments, and an individual apex segment). Artifact-containing segments in each patient were identified by two attending radiologists.

## Results

**Figure 2:**
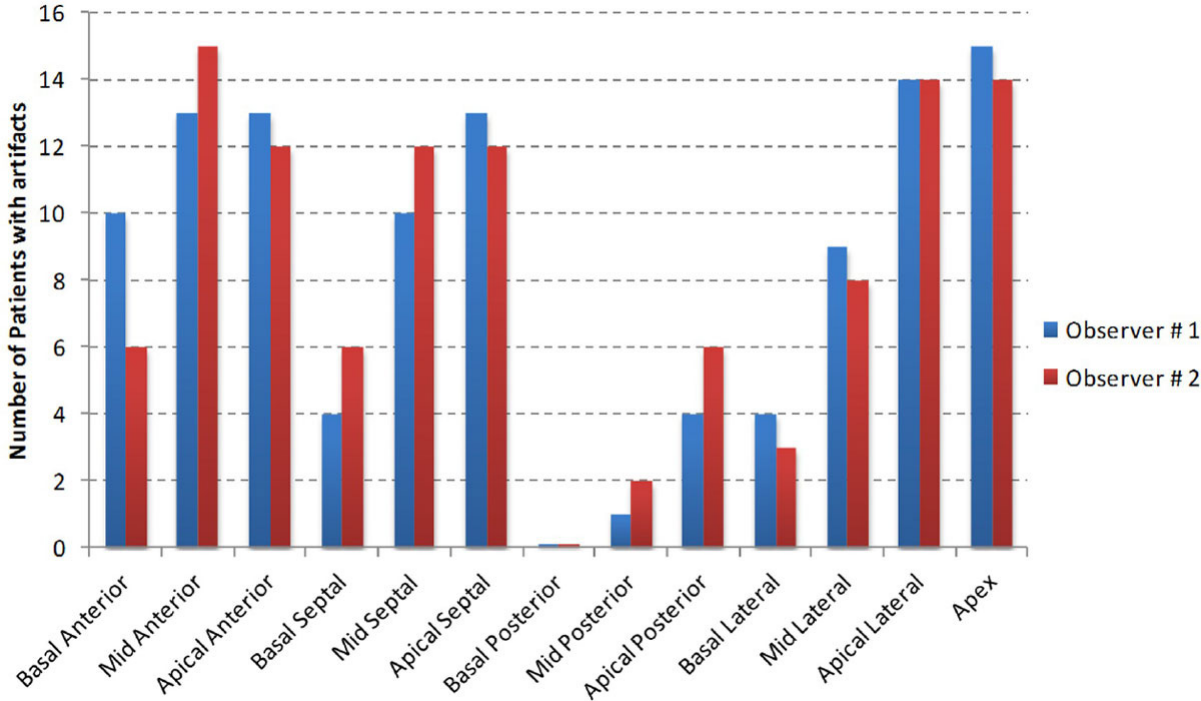
**Graph showing the number of patients who presented artifacts in each of the 13 segments of the left ventricle in the conventional LGE images**. The 3 segments that had the highest occurrence of artifacts are the apex, the apical lateral segment and the mid-ventricular anterior segment.

No HI artifacts were present in the conventional LGE images of the 3 PM patients included in the study, as well as 2 ICD patients owing to large distance of the ICD from the heart. In the remaining 20 ICD patients, HI artifacts were present in 5.6 ± 2.4 segments per patient in the conventional LGE images. All artifacts were completely eliminated in the wideband LGE images. Figure 1 shows examples of LGE images from the conventional and wideband LGE technique. Figure 2 shows the number of patients that had HI artifacts in each of the 13 segments. The three segments with the largest number of artifacts are the apex, the apical lateral and the mid-ventricular anterior segment.

## Conclusions

We have developed a wideband LGE technique to eliminate the HI artifacts seen in LGE MRI of patients with ICDs. This technique was implemented at two centers and successfully evaluated on 25 patients, leading to prominent reduction of the HI artifacts. The wideband LGE technique may enable widespread utility of LGE MRI in patients with implanted cardiac devices, in whom LGE MRI otherwise could not be used for diagnosis.

## Funding

NIH 1R21 HL118533
